# Resilience analysis and improvement strategy of microgrid system considering new energy connection

**DOI:** 10.1371/journal.pone.0301910

**Published:** 2024-04-18

**Authors:** Yongrong Zhou, Yan Zhao, Zhaoxing Ma

**Affiliations:** 1 State Key Laboratory of Technology and Equipment for Defense against Power System Operational Risks, Nanjing, Jiangsu, China; 2 Nari Technology Co., Ltd., Nanjing, Jiangsu, China; 3 School of Information and Control Engineering, Qingdao University of Technology, Qingdao, China; Wuhan University, CHINA

## Abstract

With the increasing demand for electricity, microgrid systems are facing issues such as insufficient backup capacity, frequent load switching, and frequent malfunctions, making research on microgrid resilience crucial, especially to improve system power supply reliability. This paper proposes a method for analyzing the resilience metric of new energy grid-connected microgrid system, and proposes optimization strategies to improve resilience. Firstly, a measurement method for the resilience of the microgrid system is established based on the operating characteristics of the system components. Secondly, the sensitivity relationship between system resilience and parameters is established, and an optimization model for resilience improvement strategies of microgrid systems based on parameter sensitivity is constructed. Finally, simulation verification is conducted based on the IEEE 37-node microgrid system. The results show that the proposed measurement method can scientifically and reasonably measure the resilience of the microgrid system, and the resilience improvement strategy significantly improves the operational resilience, verifying the effectiveness and robustness of the proposed analysis method.

## 1. Introduction

In recent years, the increase in electricity demand and the rapid development of various forms of energy have led to the emergence of various network structures in the power grid. Among them, microgrids are an ideal network that can not only connect to various energy sources such as cold, heat, electricity, gas, and energy storage, but also have the characteristics of flexibility and easy control. By implementing appropriate energy management and control measures, the coordination and optimization of power supply, network, load, and energy storage within the region can be achieved. However, with the increasing number of microgrid configurations and the frequent occurrence of various disasters, multiple power outages have occurred both domestically and internationally, causing huge economic losses and posing significant challenges to the safe and reliable operation of microgrids [1–4]. Therefore, studying the resilience analysis and improvement strategies of microgrid systems can enhance the implementation of functions such as renewable energy access, providing efficient energy services for users, and optimizing energy utilization within the region, and improve the resilience of microgrid systems to external disturbances. In the face of increasingly severe challenges, the academic and engineering communities at home and abroad have gradually attached importance to the study of resilience issues in microgrid systems. Against this backdrop, the concept of power system resilience emerged and achieved some good results [5–10].

The research on the measurement of power system resilience mainly focuses on evaluating indicators, including analyzing the factors that affect system resilience and evaluating the performance of the system during resilience deformation. Currently, the concept of "resilience ladder" is widely used in research, with a basic indicator set consisting of system performance degradation degree, system performance degradation speed, and system resilience process recovery time [11–13]. The resilience indicators are divided into static and dynamic resilience indicators. Static indicators typically focus on the structural level of the system, using methods such as probability theory [14] and graph theory [15] to analyze and quantify inherent properties such as the vulnerability of key nodes and the connectivity of the topological structure of the system. Dynamic indicators consider the temporal variation process of extreme events and are used to quantify the real-time response capability of the system under extreme events. Generally, dynamic indicators are constructed from the perspective of the rate of decline, minimum functional level, and recovery time under the system function curve. The literature [15] considers the structure, network connections, and services, quantifies the energy provided, and proposes a probabilistic evaluation of the resilience of the power distribution system under different weather conditions. The literature [16] establishes a simplified model of functional loss, linearizes the resilience curve into a "triangle", and then solves it to complete the analysis of system resilience. The literature [17, 18] uses the area of load loss in the resilience process as an indicator for evaluating system resilience. Reference [19] proposed a method for evaluating the resilience of multiplexing networks by improving the way connection links are added to enhance network resilience. An agent-based approach is proposed in literature [20], reinforcement learning was used to improve the recovery strategy of network systems, and the reliability of the system was optimized.

**Fig 1:**
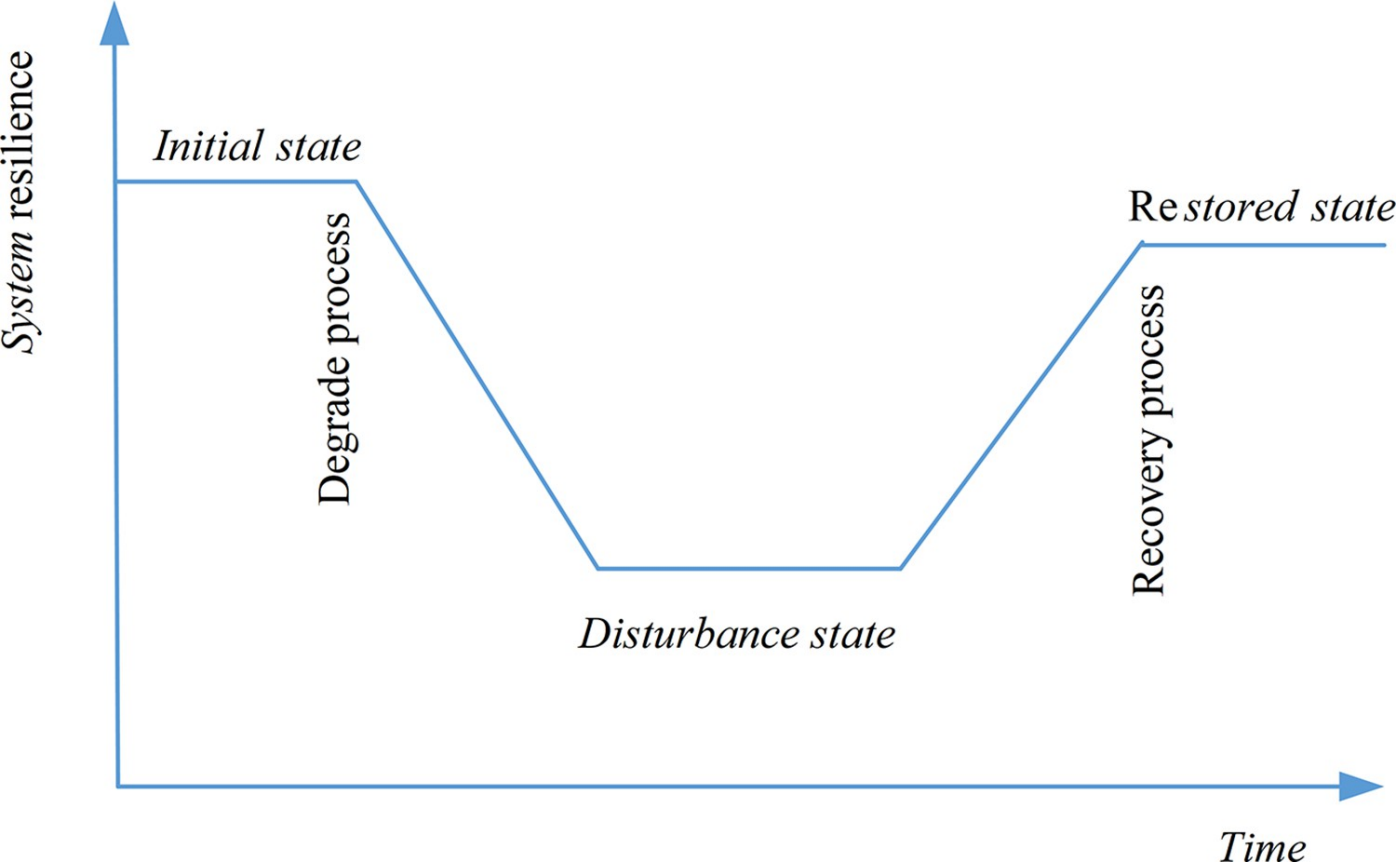
The process of resilient development.

The above method deepens the understanding of power system resilience analysis. The application of resilience index method is simple, dividing the development process of network system resilience into three states and two processes (as shown in Fig 1). Although qualitative research can be conducted on system resilience, it is difficult to construct the functional curve of the system when facing actual power systems, and quantitative analysis of the functional curve cannot be carried out. This brings difficulties to the practical application of power network system resilience measurement, which in turn affects the practicality and accuracy of system resilience measurement. In response to these shortcomings, a new resilience measurement analysis method for network systems is proposed in the article. The method idea is: the manifestation of the impact of system disturbance on the system is called fault, and the performance recovery process is called fault repair process. From fault occurrence to fault repair process, it will involve changes in the corresponding components of the system, mainly the parameters of the components. Therefore, this article uses the parameters of the components to quantify the resilience development process of the network system. Combining with the engineering application background, a detailed measurement analysis model is provided (as shown in formula (4) below), as described in section 2 below. This method overcomes the shortcomings of existing methods that are difficult to perform quantitative analysis. The method described in this article can not only reasonably describe the resilient development process of network systems, but also effectively quantify system resilience, making it easy for practical calculations and applications.

Generally speaking, the methods for measuring the resilience of power network systems mainly focus on two aspects: ①positive analysis of the factors that affect resilience; ②Evaluate the changes in the performance of the system’s resilient deformation process. The first aspect is mostly achieved through probabilistic analysis of influencing factors, such as in reference [14], which considers structure, network connectivity, and services and proposes probability assessment methods for distribution network resilience under different weather conditions. The evaluation method for the second aspect is mostly based on the concept of "resilience trapezoid", with the degree of system performance degradation, degradation rate, duration of resilience process, and speed of resilience recovery as the basic indicator set. Then, the area of the resilience trapezoid is combined with the recovery time index to measure resilience. According to the definition of system resilience, how to intuitively describe the degree of system resilience development and how to quickly recover should be the focus of resilience measurement analysis. Therefore, the area of resilience trapezoid is not the most intuitive and effective resilience measurement indicator. Literatures [17, 21] used the area of system load loss during the process of resilience development as an indicator for measuring system resilience. Literature [22] considered the ratio of the performance area of a disaster affected system to the performance area of a normal system, and established an resilience index that considers events, taking into account the time required for system recovery. The above indicators are to some extent limited by the combination of trapezoidal area and time indicators. On the basis of analyzing the resilience development process of microgrid systems, this article establishes a model to measure system resilience by utilizing the component parameters that affect the resilience development of network systems. This model not only intuitively reflects the factors that affect system resilience, but also reflects the magnitude of the impact of different factors on system resilience, laying the foundation for the design and regulation of microgrid systems.

At present, most of the strategies and methods for improving the resilience of microgrid systems rely on the application of a large amount of configuration resources, such as energy storage site selection and capacity adjustment, load switching, demand side response, grid reconstruction, etc [23]. There are various types of microgrid power sources, random loads, and complex grids, making the practical application of many strategies difficult. Literature [24] utilized active islanding to enhance the resilience of the power grid, while literature [25] applied faster response of transmission line protection systems to enhance the resilience of transmission lines. The above strategy had a certain effect on improving the resilience of the power grid, but it required a high investment cost and is difficult to implement. On the basis of analyzing the component parameters that affect the resilience of network systems, this article proposes an optimization model for resilience enhancement. This model optimizes the parameters of each component and achieves the goal of improving the resilience of network systems by adjusting the component parameters. The strategy proposed in the article avoids large-scale investment costs, has good economic efficiency, and is simple and practical.

The above-mentioned methods for enhancing the resilience of microgrids mainly adopt the idea of emergency control, which involves replanning the operation mode of the microgrid after a failure occurs and establishing an island microgrid to supply power to the load. However, there may be significant power imbalance during the establishment of the island microgrid, which may cause safety constraints such as unit climbing to exceed limits when the microgrid adjustment capability is insufficient, resulting in the failure of the island microgrid to establish safely and stably. In addition, due to the power flow on the transmission line before the failure, the sudden change in power on the line after the failure can cause a significant step-type power impact on the island microgrid [26], which may lead to frequency exceeding limits in the island microgrid, resulting in power cut protection actions and the collapse of the island microgrid. These will limit the effectiveness of resilience enhancement strategies for microgrid systems.

In summary, there are still some gaps that need to be filled in previous research. Unlike previous studies that typically focus on system resilience indicators or optimization scheduling problems, this article focuses on studying resilience measurement methods and strategies to enhance resilience in power network systems, and proposes qualitative and quantitative analysis of resilience measurement methods.

Addressing the shortcomings of the above methods in analyzing microgrid resilience and improving strategies, this paper proposes a resilience analysis and improvement strategy for microgrid systems. Due to the significant uncertainty of disturbances experienced by network systems, and the diversity of performance changes after disturbances, it is not sufficient to reflect the performance of the entire process from disturbance to repair if the network system resilience is determined solely or described solely through inherent parameters. Therefore, based on the theory of resilience engineering, this article adopts reliable parameters of network systems, considers the entire process and uncertainty of system performance changes, and conducts resilience measurement analysis on network systems. This article analyzes the impact of disturbances on the resilience of microgrid systems containing multiple components, and constructs an evaluation system for measuring the resilience of microgrid systems. Based on this, the sensitivity analysis method of system parameters was used to study the influence of various parameters on system resilience, and then the parameters can be adjusted to change the system resilience, achieving the goal of improving system resilience. In this study, an analysis method for the importance of each component in a network system is proposed, and the different effects of the importance of each component on system resilience are studied. The main research contents include: 1) Taking a simple microgrid system as an example, this paper compares and analyzes the methods of measuring network resilience, explains the characteristics of different methods, and based on this, proposes a new resilience measurement method for microgrid system. 2) By sensitivity calculation, the relationship between component related parameters and system resilience is constructed, and the degree of influence of different components on resilience in the system is analyzed. An optimization model for improving system resilience is established. 3) Taking a microgrid system with multiple new energy connections as a simulation example, multiple operating conditions are set, and the effectiveness of the proposed resilience measurement method and optimization improvement strategy is analyzed and verified through calculation.

The chapter arrangement of this article is as follows. Section 1 introduces some existing methods for analyzing system resilience and compares their respective characteristics. At the same time, the basic idea of studying network system resilience analysis in this article is introduced. Section 2 elaborates on the new problems and requirements faced by microgrid system resilience. Section 3 introduces the established resilience analysis framework and presents the resilience measurement analysis method for a simple microgrid system. Section 4 analyzes the resilience measurement of microgrid systems containing new energy, and also expounds the resilience improvement strategies for microgrid systems. In section 5, it presents the constraint model for resilience analysis of microgrid systems. Section 6 presents the simulation examples and microgrid models, and verifies the effectiveness of the proposed resilience analysis method and improvement strategies through simulation examples. Section 7 concludes this paper with a summary and suggests possible direction for future research.

## 2. New issues and requirements for the resilience of microgrid systems

Microgrid, as an important form of power energy supply in the power system, plays an important role in reducing carbon emissions, improving the flexibility and economy of power supply, and contributing to the reliable operation of the power system. In recent years, extreme weather and large-scale faults have occurred frequently, seriously affecting the reliability of power network operation. Therefore, it is urgent to improve the resilience of power network operation to effectively respond to extreme events, minimize losses, and accelerate system power recovery.

Microgrids contain various forms of energy, such as traditional thermal power generation, wind power, photovoltaic power generation, etc. The microgrid is flexible in networking, operates in various ways, and has a high degree of power electronics, resulting in complex nonlinear and time-varying characteristics of the system. In addition, the intermittency of new energy output and the randomness of load side participation reduce the adaptability between sources and loads. Meanwhile, in recent years, with the rapid development of new technologies and new energy generation, microgrid systems have shown characteristics such as high proportion of new energy, high degree of power electronicization, and high degree of freedom in load energy consumption, further enhancing the resilience of microgrid systems to cope with extreme events. Microgrid systems urgently need to meet the transition from passive defense to effective response, catering to the demands and challenges of improving the resilience of microgrid systems.

The requirement for improving the resilience of microgrid systems is based on traditional networks, mainly expanding and improving from the process of fault occurrence. Before a malfunction occurs, accurately identify the parameters related to resilience enhancement in the microgrid system and reinforce important links. The structural characteristics and operation mode of microgrids have shaped a new form of network. By efficiently identifying key component parameters, resource deployment before faults is achieved, ensuring system robustness. Microgrid systems fully tap into the resource resilience potential of different components in the system and mobilize the system’s rapid recovery capability. After a fault occurs, the network system will suffer varying degrees of damage, and the damaged components and network system performance urgently need to be repaired. Fully exploring the importance of different components for system resilience recovery, effectively aggregating and coordinating them, cooperating with the component repair process, and formulating optimized microgrid system resilience recovery strategies are crucial for reducing load outage time and improving the efficiency of unit and load recovery.

## 3. Analysis of resilience metrics for simple microgrid systems

In the operation of a microgrid, system resilience is manifested as the process of recovering from disturbances to the system [27]. System recovery can be measured from two perspectives: 1. From the perspective of the system, through task reorganization, the system has the ability to meet new operating requirements; 2. From the perspective of system components, through the operation and maintenance personnel’s repair of the faulty components, it can be restored to a new state and meet the operating requirements. This article will describe the resilience development process from the perspective of system components, applying component reliability-related theories, from component failure to repair, and then the system returns to a new operating state. That is, the component is affected by disturbances and has an impact on the operation of the microgrid system, mainly manifested as failure, where the performance recovery process is fault repair. In the evaluation of system resilience in a microgrid system, the intermittency of the output power of new energy sources will also be considered, which will affect the resilience recovery of the system.

**Fig 2:**
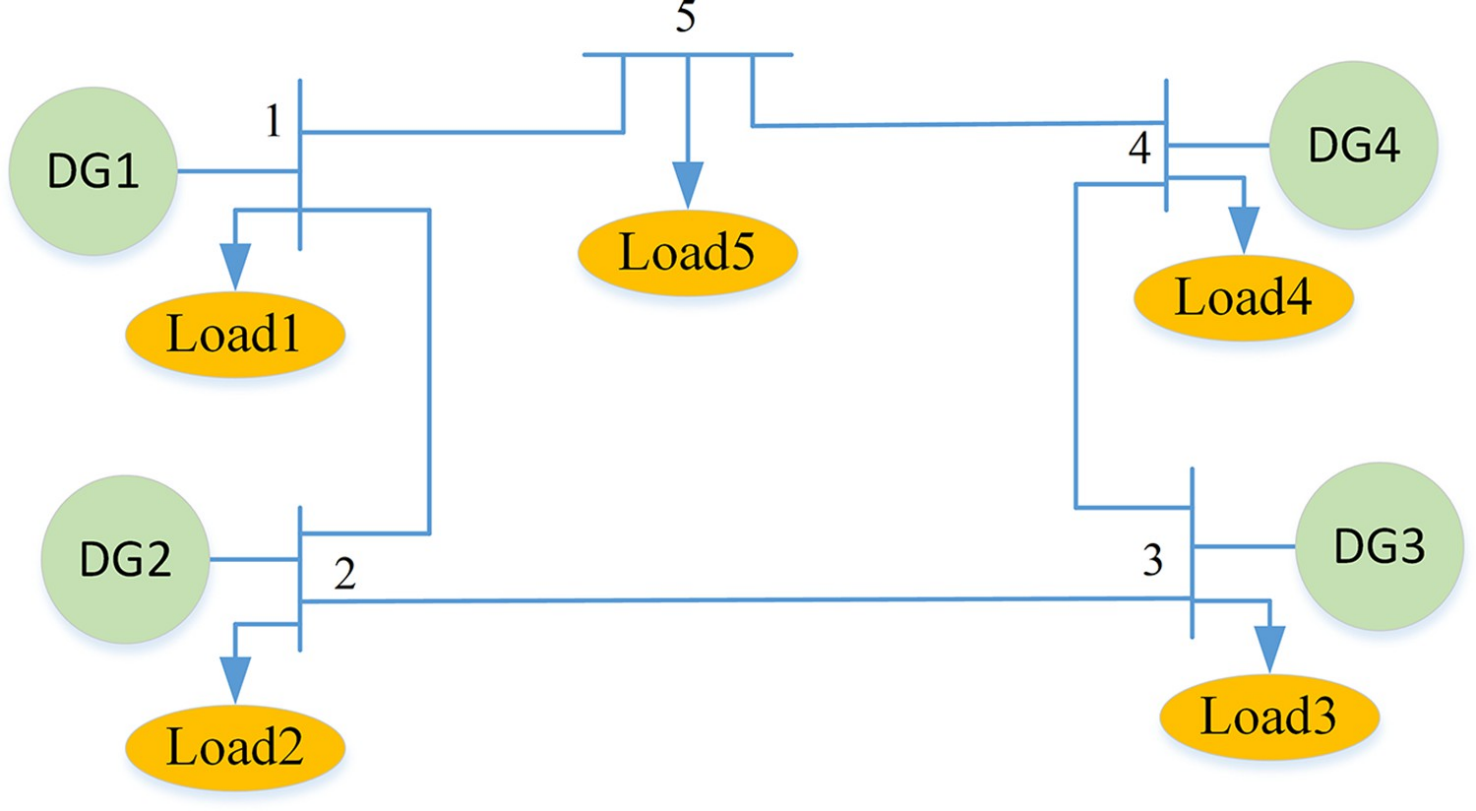
Simple microgrid system program.

According to the complexity of the structure, microgrid systems can be divided into simple microgrids and complex microgrids. Simple microgrids contain fewer components and have a simpler structure [28], and the topology is shown in Fig 2. This simple microgrid system contains 4 power injection points and 5 load nodes. Complex microgrid systems contain more power points, lines, loads, and other components, and the network structure is more complex. This section will start with simple microgrid systems to analyze the resilience evaluation methods.

For the microgrid system containing intermittent new energy, the resilience evaluation of the system needs to consider the continuously changing performance of each component, the intermittency of the power output of new energy generation, the probability of component failure, and the uncertainty of fault repair. If only a single deterministic or probabilistic resilience measurement method is used, the accuracy will be difficult to guarantee.

Considering the performance of system components, the resilience metric *E*_*s*_ of the system can be expressed as follows [29]:

$$E_{s}=\sum_{e_{i}\in E}P_{i}\int e_{i}dt$$ (1)

Where *e*_*i*_ is the performance change curve of component *i* after the system is perturbed; *P*_*i*_ is the probability of the corresponding curve; *E* is the set of performance change curves of each component in the system.

**Fig 3:**
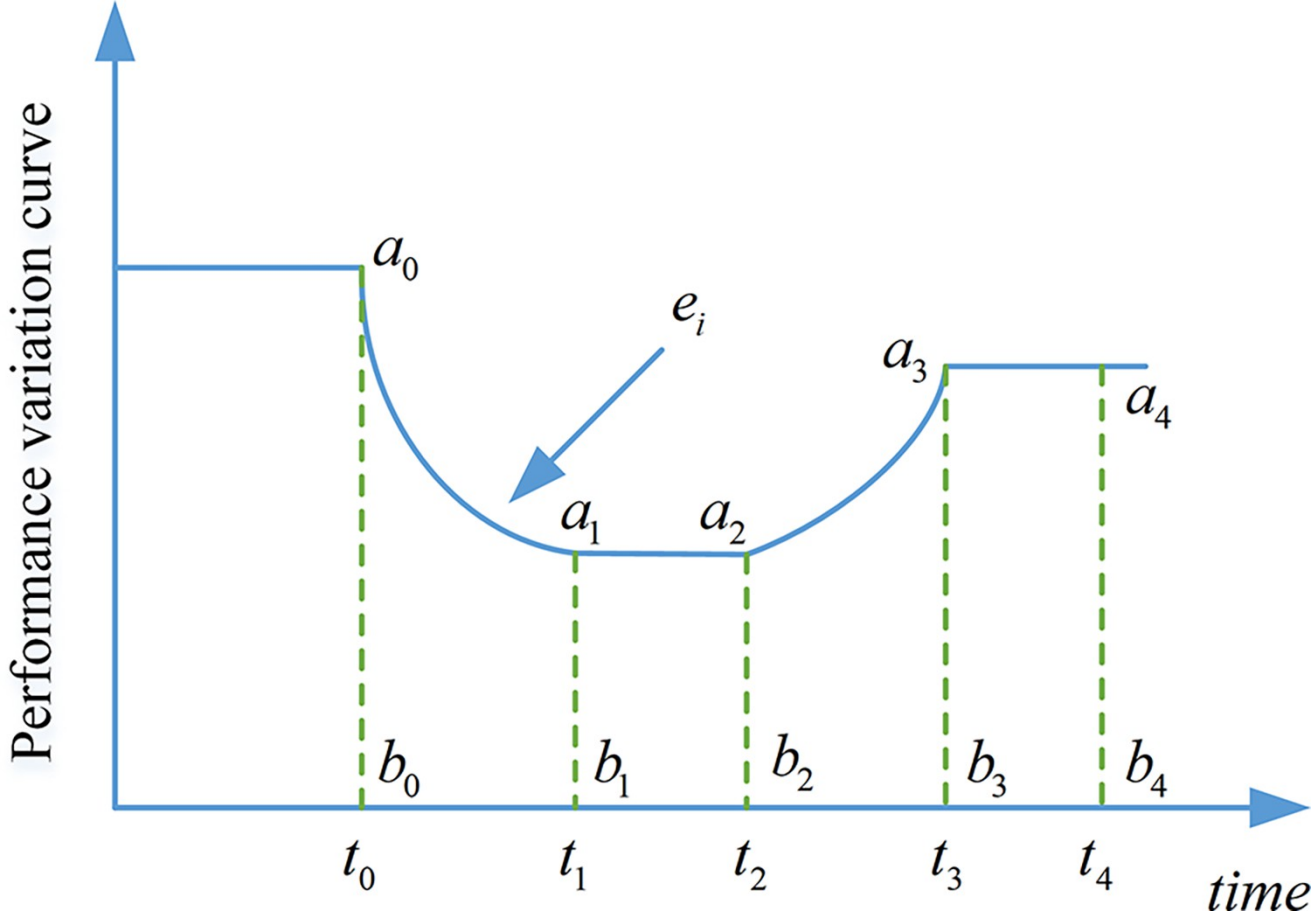
Performance variation curve of component *i*.

After the microgrid system is disturbed, the performance of component *i* meets the "bathtub curve", and its variation curve can be represented in Fig 3.

In Fig 3, after being subjected to a disturbance, the performance of the microgrid system component *i* returns to a new operating state after a period of operation and maintenance management. Its resilience can be obtained from the performance variation curve. The resilience variation process of the microgrid system can be expressed by the following formula.

$$S_{T}=\frac{\sum_{f_{i}\in F_{J}}\left(f_{1}+f_{2}+f_{3}+f_{4}\right)}{t_{4}-t_{0}}$$ (2)

Where *S*_*T*_ is resilience for the system; *F*_*J*_ is a collection of curves for the performance degradation of system components; *f*_*i*_ (*i =* 1,2,3,4) is the expected values of the performance curve in the four stages of performance degradation, performance maintenance, performance recovery, and post-recovery; *t*_0_ is the starting point of disturbance; *t*_1_ is the end of the performance decline; *t*_2_ is the time when its performance began to recover; *t*_3_ is the time when it completes the performance recovery; *t*_4_ is the time after it completes the performance recovery. As shown in Fig 2. The calculation of each variable in Eq (2) is represented by the following equation.

$$f_{1}=P_{1}(f)P_{2}(q_{2}|f)\int_{t_{0}}^{t_{1}}q_{2}(t)dt$$ (3A)

$$f_{2}=P_{1}(f)P_{2}(q_{2}|f)P_{3}(q_{3}|f,q_{2})\int_{t_{1}}^{t_{2}}q_{2}(t)dt$$ (3B)

$$f_{3}=P_{r}(f)P_{3}(q_{3}|f,q_{2})\int_{t_{2}}^{t_{3}}q_{3}(t)dt$$ (3C)

$$f_{4}=P_{r}(f)P_{3}(q_{3}|f,q_{2})\int_{t_{3}}^{t_{4}}q_{3}(t)dt$$ (3D)

Where *P*_1_(*f*) is the probability of disturbance *f* in the microgrid system; *P*_*r*_(*f*) is the recovery rate of the microgrid system under the disturbance *f*; *P*_2_(*q*_2_│*f*) is the conditional probability of the performance degradation curve of the microgrid system under the condition of perturbation *f* being *q*_2_(*t*); *P*_3_(*q*_3_│*f*, *q*_2_) is the conditional probability of the performance recovery curve of the microgrid system under the condition of a performance reduction of *q*_2_ under perturbation *f*, with a performance recovery curve of *q*_3_.

Traditional network system resilience assessment method by considering the characteristics of the "bathtub curve" in a certain scenario and the probability of each scenario occurring. The calculation process is shown in Eq (1)—(3) above. In general, it is difficult to obtain the performance change curve of microgrid system components. The bathtub curve is more suitable for qualitative analysis of the resilience development process of microgrid systems, but there are difficulties in quantitative analysis. In this study, the influence of system component parameters on the resilience of microgrid systems is considered. After the microgrid system was disturbed, the probability of component failure is taken into account, and the repair time under disturbed conditions is considered in the component repair speed model. The impact of component reliability on system resilience is considered, taking into account the interval between component failures. When the microgrid system is disturbed by faults, the above factors can comprehensively and truthfully reflect the system’s resilience and recovery ability. Therefore, to measure the resilience of microgrid systems, this paper proposes an resilience measurement model that is convenient for computational applications, as shown below:

$$S_{w}=\sum_{i\in \Omega }wt_{i}[\left(1-P_{f}^{i})+\frac{1}{T^{i}}P_{f}^{i}(P_{r}^{i}(x_{1}^{i}T_{1}^{i}+x_{2}^{i}T_{2}^{i})+(1-P_{r}^{i})x_{1}^{i}T^{i}\right)]$$ (4A)

$$wt_{i}=\frac{S_{pi}}{S_{\sum }}$$ (4B)

Where Ω is the collection of components contained in the microgrid; *wt*_*i*_ is the weight of component *i*; *S*_*pi*_ is the power of component *i*; *S*_*Σ*_ is the total power of the system; $P_{f}^{i}$ is the failure rate of the microgrid component *i*; $P_{r}^{i}$ is the repair rate of microgrid component *i;* $x_{1}^{i}$ is the performance of component *i* after the microgrid failure after normalization; $x_{2}^{i}$ is the performance of component *i* after normalization and fault repair; $T_{1}^{i}$ is the average time for repairing component *i*; $T_{2}^{i}$ is the average time between component *i* malfunctions; *T*^*i*^ = $T_{1}^{i}+T_{2}^{i}$.

## 4. Resilience analysis of microgrid system with new energy integration

Microgrid systems with more intermittent renewable energy generation have a large number of components and complex operating characteristics. The failure rate, repair rate, mean time between failures, mean time to repair, and other factors of each component, including the characteristics of intermittent renewable energy generation, jointly constitute the parameter system of resilience metrics for microgrid systems.

### 4.1. Strategies to enhance the resilience of microgrid systems

**Fig 4:**
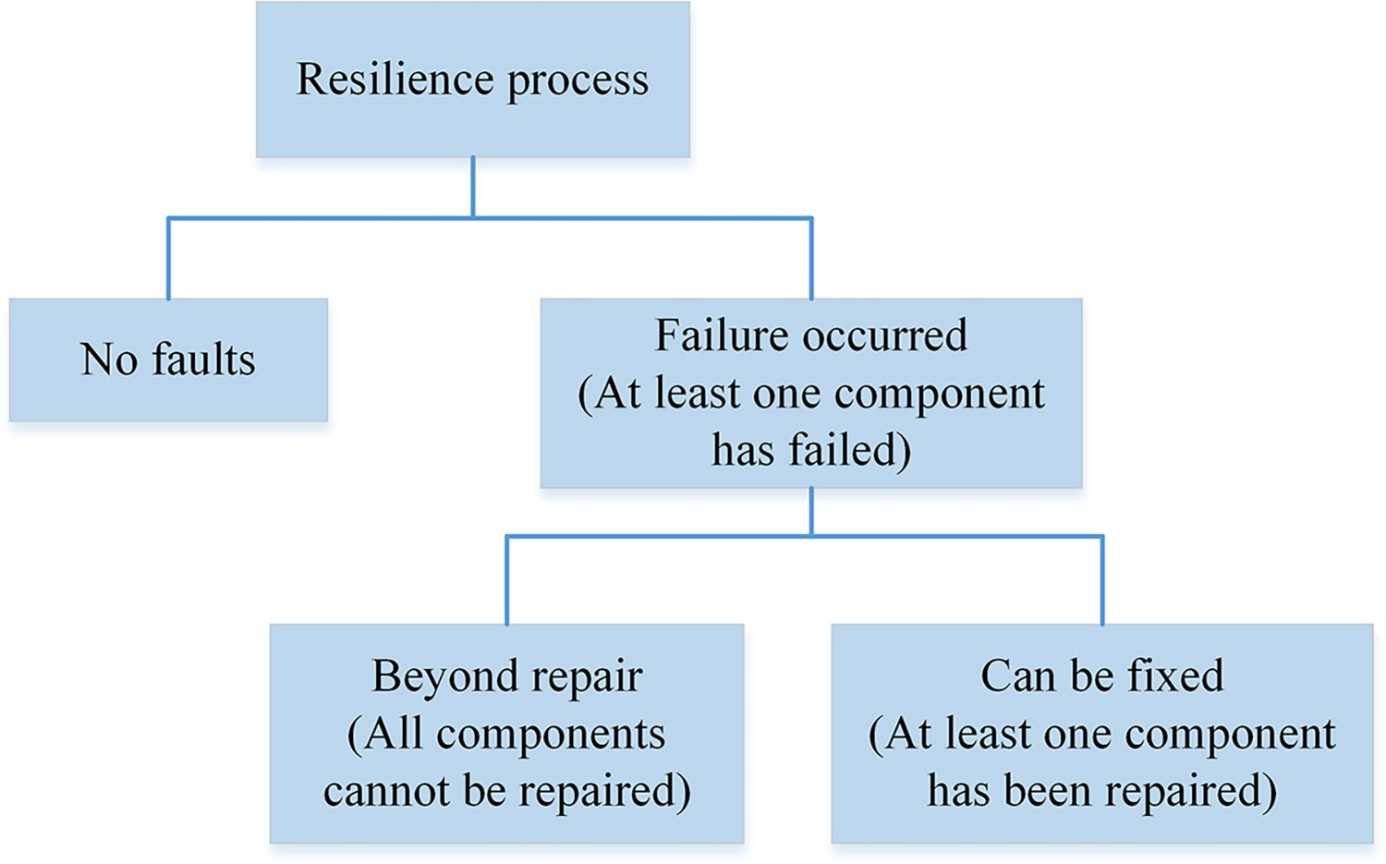
The resilient development process of microgrid system.

The resilience development process of the microgrid system components after failure can be represented as shown in Fig 4 below.

The expression of the resilience metric of the microgrid in Eq (4) shows that the parameters that affect the resilience of the microgrid are: the failure rate $P_{f}^{i}$ of component *i*, the repair rate $P_{r}^{i}$ of component *i*, the performance $x_{1}^{i}$ after component *i* fails, the performance $x_{2}^{i}$ after component *i* is repaired, and the time *T*^*i*^ of component *i*. This article uses sensitivity analysis to analyze the strategies for improving the resilience of the microgrid system under the condition of changing one parameter. The first-order sensitivity of each parameter in Eq (4) is expressed as follows.

$$\frac{\partial S_{w}}{\partial P_{f}^{i}}=\frac{1}{T^{i}}[P_{r}^{i}(x_{1}^{i}T_{1}^{i}+x_{2}^{i}T_{2}^{i})+(1-P_{r}^{i})x_{1}^{i}T^{i}]-1$$ (5A)

$$\frac{\partial S_{w}}{\partial P_{r}^{i}}=\frac{1}{T^{i}}P_{f}^{i}(x_{1}^{i}T_{1}^{i}+x_{2}^{i}T_{2}^{i}-x_{1}^{i}T^{i})$$ (5B)

$$\frac{\partial S_{w}}{\partial x_{1}^{i}}=\frac{1}{T^{i}}P_{f}^{i}[P_{r}^{i}T_{1}^{i}+(1-P_{r}^{i})T^{i}]$$ (5C)

$$\frac{\partial S_{w}}{\partial x_{2}^{i}}=\frac{1}{T^{i}}P_{f}^{i}P_{r}^{i}T_{2}^{i}$$ (5D)

$$\frac{\partial S_{w}}{\partial T_{1}^{i}}=P_{f}^{i}P_{r}^{i}\frac{(x_{1}^{i}-x_{2}^{i})T_{2}^{i}}{{\left(T_{1}^{i}+T_{2}^{i}\right)}^{2}}$$ (5E)

$$\frac{\partial S_{w}}{\partial T_{2}^{i}}=-P_{f}^{i}P_{r}^{i}\frac{(x_{1}^{i}-x_{2}^{i})T_{1}^{i}}{{\left(T_{1}^{i}+T_{2}^{i}\right)}^{2}}$$ (5F)

Wherein, the physical meaning of each parameter in the formula is as described in formula (4).

**Fig 5:**
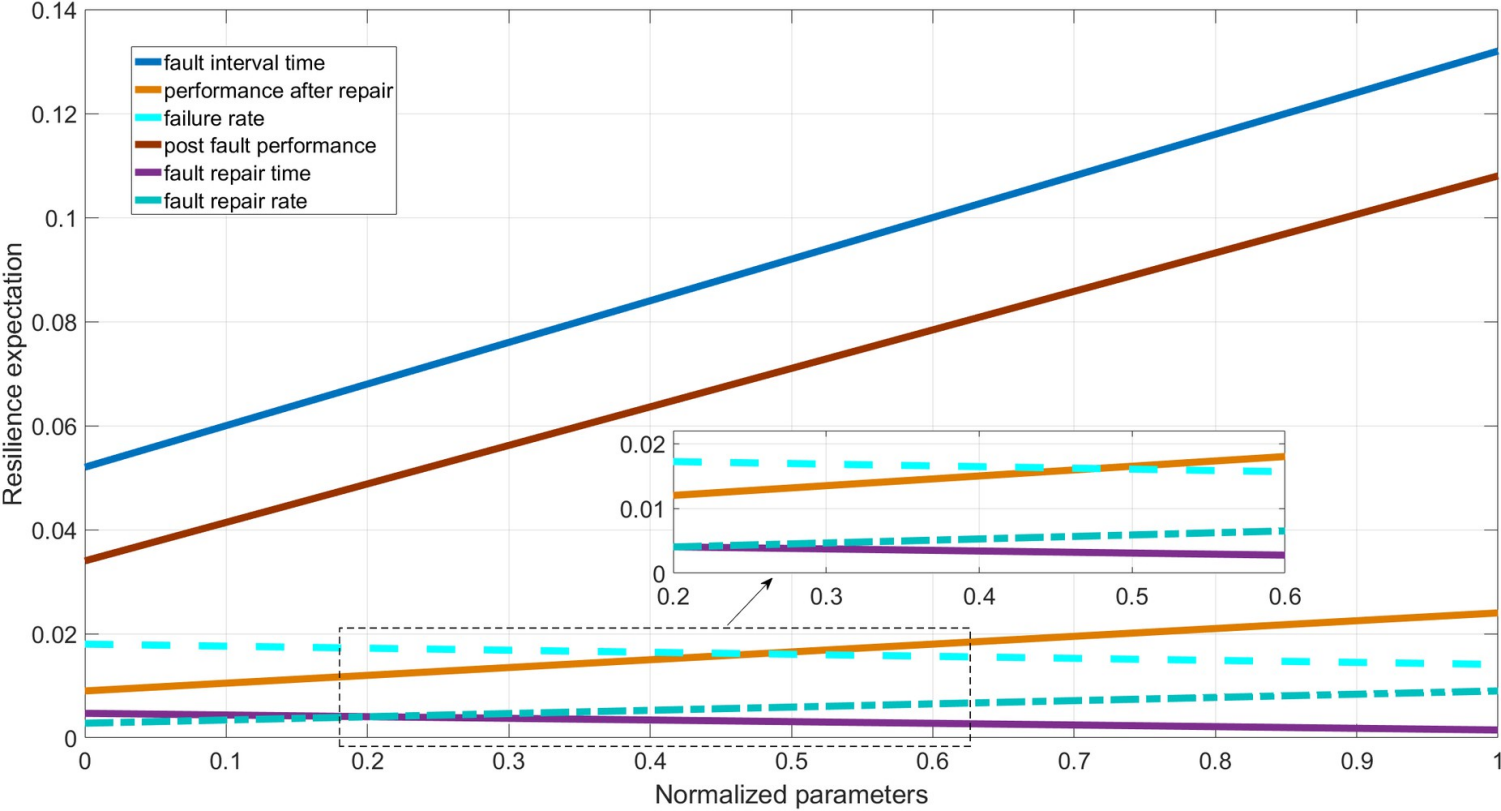
Performance resilience change after parameter sensitivity repair of components.

The calculation results of Eq (5) show that the value of the first-order sensitivity varies with different parameters. The larger the value of the first-order sensitivity, the greater the impact of the parameter on the resilience of the microgrid; conversely, the smaller the value. Therefore, when formulating optimization strategies to improve the resilience of the microgrid system, priority should be given to the component parameters that have the greatest impact on the resilience of the microgrid, and then to the component parameters that have the least impact on the resilience of the microgrid. Taking the simple microgrid system shown in Fig 2 as an example, a calculation analysis is conducted. In the system shown in Fig 2, there are four power nodes and five load nodes. Now calculate the first-order sensitivity of the parameters contained in node 3. The calculation results are shown in Fig 5.

In Fig 5, the abscissa is the normalized parameter, and the ordinate is the sensitivity of the system resilience with respect to the corresponding parameter. From Fig 5, it can be seen that the sensitivity corresponding to the fault repair rate of the microgrid system component is the largest, that is, when the microgrid component fails, improving the fault repair rate is the most effective way to enhance the resilience of the microgrid. The second is the performance of the system after fault repair. The sensitivity of fault repair time is negative, that is, when the component fails, the longer the fault repair time, the more detrimental it is to the improvement of the resilience of the microgrid system. This trend is consistent with the influence of various parameters of the microgrid system components on the system resilience.

From the above analysis, it can be concluded that when designing and analyzing microgrid systems, the focus should be on improving the parameters that maximize system resilience, and finally considering the parameters that have the least impact on resilience. The optimization control strategy for component parameters proposed in the article enhances the resilience of microgrid systems while also enhancing their operational performance:

1) Improving the repair rate of components enhances the recoverability of microgrid systems;

2) Maintaining good performance of microgrid systems after failures is equivalent to enhancing their ability to resist disturbances and faults, and improving their robustness;

3) After the fault repair of the microgrid system, its performance can be restored to a sufficiently high level, reflecting the good resilience of the microgrid system. This fast recovery ability also reflects the good recoverability of the microgrid system;

4) Maintaining a sufficiently small failure rate and interval time for microgrid systems demonstrates their good recoverability and reliability. This kind of recoverability and reliability is an important goal in the design and maintenance of microgrid systems, and it is also the key to improving the resilience of microgrid systems.

From theoretical analysis and computational results, it can be seen that adjusting parameters within a certain range can effectively improve the resilience of microgrid systems. This article applies mathematical optimization methods, combined with analytical sensitivity relationships, to provide optimal strategies for improving the resilience of microgrid systems. Mathematical optimization model decision-making is mainly determined by the selection of variables and the calculation method of the system and objective function under study. This section presents an optimization model for improving the resilience of microgrid systems, as shown below.

$$\left\{ \begin{array}{l} \max(S_{w}+\frac{1}{Ec})\\ s.t.f(x)=0\\ \;h{\left(y\right)}_{\min}\leq h(y)\leq h{\left(y\right)}_{\max} \end{array}\right.$$ (6A)

$$Ec=\sum_{j\in \Omega_{0}}c(\vartheta^{j})\vartheta^{j}$$ (6B)

Where *S*_*w*_ is the resilience measure of microgrid systems, as described in Eq (4); *Ec* represents the economic feasibility of adjusting system parameters; *ϑ*^*j*^ is the j-th parameter value; *c*(*ϑ*^*j*^) is the corresponding cost; Ω_0_ is the parameter set; *f*(*x*) is the power flow equation of the microgrid system, as described in Eq (13) below; *h*(*y*) is a variable inequality constraint, as described in Eq (14) below, *h*(*y*)_max_、*h*(*y*)_min_ represents the upper and lower limits respectively.

The formula (6) is a non-linear, non-convex optimization mathematical problem, which constitutes a multi-objective optimization space after adding sensitivity constraints. For the microgrid system, it is known from Eq (5) that the same component contains multiple parameter sensitivities. When the corresponding parameter sensitivity is less than 0, increasing the parameter will reduce the resilience of the microgrid; when the corresponding parameter sensitivity is greater than 0, increasing the parameter will enhance the resilience of the microgrid. Based on the above analysis, this article proposes an optimization strategy for improving the resilience of microgrid systems based on the Pareto optimization algorithm, with the resilience measurement formula and economy of microgrid systems as the objective functions. Under corresponding constraints, regulate the parameters of system components to achieve the goal of maximizing system resilience. At the same time, sensitivity conditions are added to the objective function, and the resilience optimization model of the microgrid system is represented as follows:

$$\left\{ \begin{array}{l} \max(S_{w}+\frac{1}{Ec})\\ s.t.f(x)=0\\ \;h{\left(y\right)}_{\min}\leq h(y)\leq h{\left(y\right)}_{\max}\\ \;\tau_{\min}^{i}\leq \tau^{i}\leq \tau_{\max}^{i}\\ \;g(\tau^{i})\geq 0 \end{array}\right.$$ (7A)

$$Ec=\sum_{j\in \Omega_{0}}c(\vartheta^{j})\vartheta^{j}$$ (7B)

$$g(\tau^{i})=(-\frac{\partial S_{w}}{\partial P_{f}^{i}},\frac{\partial S_{w}}{\partial P_{r}^{i}},\frac{\partial S_{w}}{\partial x_{1}^{i}},\frac{\partial S_{w}}{\partial x_{2}^{i}},-\frac{\partial S_{w}}{\partial T_{1}^{i}},\frac{\partial S_{w}}{\partial T_{2}^{i}})$$ (8)

Where $\tau^{i}=(P_{f}^{i},P_{r}^{i},x_{1}^{i},x_{2}^{i},T_{1}^{i},T_{2}^{i})$, $\tau_{max}^{i}$, $\tau_{min}^{i}$ represents the upper and lower limits of the control parameters, respectively; $\frac{\partial S_{w}}{\partial P_{f}^{i}}$, $\frac{\partial S_{w}}{\partial P_{r}^{i}}$, $\frac{\partial S_{w}}{\partial x_{1}^{i}}$, $\frac{\partial S_{w}}{{\partial x}_{2}^{i}}$, $\frac{\partial S_{w}}{\partial T_{1}^{i}}$, $\frac{\partial S_{w}}{\partial T_{2}^{i}}$ derived the sensitivity expression for each parameter, as shown in Eq (5); other parameters are described in Eq (6).

This article establishes a resilience optimization model for microgrid systems as shown in Eq (7) above, with the parameters of each component, including failure rate $P_{f}^{i}$, repair rate $P_{r}^{i}$, post-failure performance $x_{1}^{i}$, post-repair performance $x_{2}^{i}$, and time *T*^*i*^, as control variables. Based on the sensitivity of each parameter, the control parameters are adjusted to achieve the goal of improving the resilience of the microgrid system.

In most optimization algorithm applications, the search for solutions is usually carried out in the decision variable space, but for multi-objective optimization algorithms, this method is not efficient. In order to improve the search efficiency of the solution, this paper uses Pareto optimization algorithm to search the solution in the objective function space. This article improves the traditional Pareto optimization method by guiding the search of decision variable space through Pareto optimization in the objective function space. The proposed sensitivity expression is the analytical mapping of Pareto optimization in the decision variable space, and the decision variable space is further divided into the original feasible domain through this constraint. Searching in the decision variable space that conforms to the Pareto optimization premise reduces the feasible domain space of the decision variables while ensuring optimal solutions, thereby improving the efficiency of the optimization model.

### 4.2. Resilience analysis of microgrid system with intermittent new energy

The microgrid system contains many components, and each component may fail or intermittent new energy generation may not achieve rated output, which will affect the resilience of the microgrid system. However, the impact of different components is not the same. Among them, new energy generation has the characteristic of intermittency. Taking wind power as an example, this paper expounds the impact of wind power output on system resilience evaluation. Due to the influence of wind speed, the output power of the wind turbine may be rated power, or it may be 0, or it may be within the range between 0 and rated power. When the output power of the wind turbine is rated, it is considered that the wind turbine is operating normally and has not failed, and the performance of the wind turbine component is 1. When the output power of the wind turbine is 0, it can be considered that the wind turbine has failed, and the performance of the wind turbine component is 0.Therefore, according to the actual output power of intermittent power generation, this paper gives the following performance expression of wind turbine components.

$$P_{fn}^{i}=\{\begin{array}{c} 0,asP_{fna}^{i}=0\\ \frac{P_{fna}^{i}}{P_{fnr}^{i}},as0<P_{fna}^{i}<P_{fnr}^{i}\\ 1,asP_{fna}^{i}=P_{fnr}^{i} \end{array}$$ (9)

Where $P_{fn}^{i}$ is the performance of intermittent energy wind turbine component *i*; $P_{fnr}^{i}$ is the rated power of the intermittent energy wind turbine *i*; $P_{fna}^{i}$ is the actual output power of the intermittent energy wind turbine *i*. The intermittent new energy studied in this article mainly includes wind power generation and photovoltaic power generation. Applying the same idea, it can obtain the performance expression of photovoltaic power generation components.

### 4.3. Identification of component importance for enhancing the resilience of microgrid systems

In a microgrid system, each component plays a different role and has different effects on the resilience of the microgrid [30, 31]. Some components are very important for enhancing the resilience of the microgrid, while others have a smaller impact on it. Clarifying the relationship between the importance of each component on the resilience of the microgrid is important for the planning and design of the microgrid system, and also provides a reference for enhancing the resilience strategy of the microgrid. This section provides an analysis method for the importance of components in the microgrid system.

Suppose that microgrid component *j* fails under a certain disturbance and cannot be repaired. The impact of this condition on the resilience of the microgrid system can reflect the importance of component *j* to the microgrid system. Therefore, this section presents an expression for measuring the importance of component *j*, denoted as *W*_*j*_, as follows.

$$W_{j}=\frac{S_{w}-S_{wj}^{\prime }}{\underset{j\in \Omega }{\max}(S_{w}-S_{wj}^{\prime })}$$ (10)

Where *S*_*w*_ is responsible for the system resilience of the microgrid during normal operation, *S΄*_*wj*_ is the resilience of the microgrid system when a microgrid component *j* fails and cannot be repaired; Ω is the set of components in the microgrid system.

After identifying the key components for improving the resilience of microgrid systems, the repair resources of the system can be tilted towards the key components, thereby suppressing the negative impact of faults on the resilience of microgrid systems and achieving the goal of improving the resilience of microgrid systems from the side.

## 5. Microgrid system model and resilience analysis

The microgrid contains a large number of distributed new energy power, and its detailed model and constraints are described as follows.

### 5.1. Microgrid system model

This article studies the resilience of microgrids, considering the impact of intermittent power on resilience metrics. The impact of intermittent power is only studied for wind power and photovoltaic power generation. The mathematical models for wind power and photovoltaic power generation are given below.

Wind power obeys Weibull distribution [17, 32], and the mathematical model of wind power generation is expressed as:

$$P_{w}^{i}=\{\begin{array}{c} 0,asv>v_{\max}orv<v_{\min}\\ P_{w}^{ie},asv^{e}<v<v_{\max}\\ k_{v}^{i}P_{w}^{ie},asv_{\min}<v<v^{e} \end{array}$$ (11)

Where $P_{w}^{i}$ is the actual output power of the *i*-th wind turbine; $P_{w}^{ie}$ is the rated output power of the *i*-th wind turbine; $k_{v}^{i}$ is the proportional coefficient of the output power of the wind turbine *i*(related to wind speed); *v* is the actual wind speed; *v*_*min*_ is the minimum wind speed for the wind turbine to cut in; *v*_*max*_ is the maximum wind speed cut for the wind turbine; *v*^*e*^ is rated wind speed.

The mathematical model of photovoltaic power generation is expressed as:

$$P_{s}^{i}=P_{se}^{i}\beta_{s}^{i}\frac{L_{s}}{L_{se}}$$ (12)

Where $P_{s}^{i}$ is actual output power of the *i*-th photovoltaic power generation; $P_{se}^{i}$ is the *i*-th photovoltaic power generation rated output power; $\beta_{s}^{i}$ is the output efficiency of photovoltaic power generation system; *L*_*s*_ is the actual light intensity; *L*_*se*_ is the light intensity under rated conditions.

### 5.2. Constraint condition

The power flow equation of the microgrid system is expressed as follows.

$$P_{i}=U_{i}\sum_{j=1}^{n}U_{j}(G_{ij}\cos\varphi_{ij}+B_{ij}\sin\varphi_{ij})$$ (13A)

$$Q_{i}=U_{i}\sum_{j=1}^{n}U_{j}(G_{ij}\sin\varphi_{ij}-B_{ij}\cos\varphi_{ij})$$ (13B)

Where *P*_*i*_、*Q*_*i*_ represents the active power and reactive power of node *i*, respectively; *U*_*i*_、*U*_*j*_ is the voltage amplitude of the node; *G*_*ij*_、*B*_*ij*_ is the conductance and susceptance of the branch, respectively; *φ*_*ij*_为is the phase angle difference of branch *i*-*j*; *n* is the number of system nodes.

The microgrid system should meet the following constraints:

$$P_{Gi,\min}\leq P_{Gi}\leq P_{Gi,\max}$$ (14A)

$$Q_{Gi,\min}\leq Q_{Gi}\leq Q_{Gi,\max}$$ (14B)

$$-P_{ij,\max}\leq P_{ij}\leq P_{ij,\max}$$ (14C)

$$U_{i,\min}\leq U_{i}\leq U_{i,\max}$$ (14D)

Where *P*_*Gi*,*max*_, *P*_*Gi*,*min*_ is the upper and lower limits of the active output of the power generation equipment *i*, respectively; *Q*_*Gi*,*max*_, *Q*_*Gi*,*min*_ is the upper and lower limits of the reactive output of the power generation equipment *i*, respectively; *P*_*ij*,*max*_ is the maximum active power of the line *i*-*j*; *U*_*i*_ is the voltage of node *i*; *U*_*i*_,_*max*_, *U*_*i*_,_*min*_ represents the upper and lower limits of voltage amplitude for node *i*, respectively.

### 5.3. Flow of resilience analysis for microgrid system

**Fig 6:**
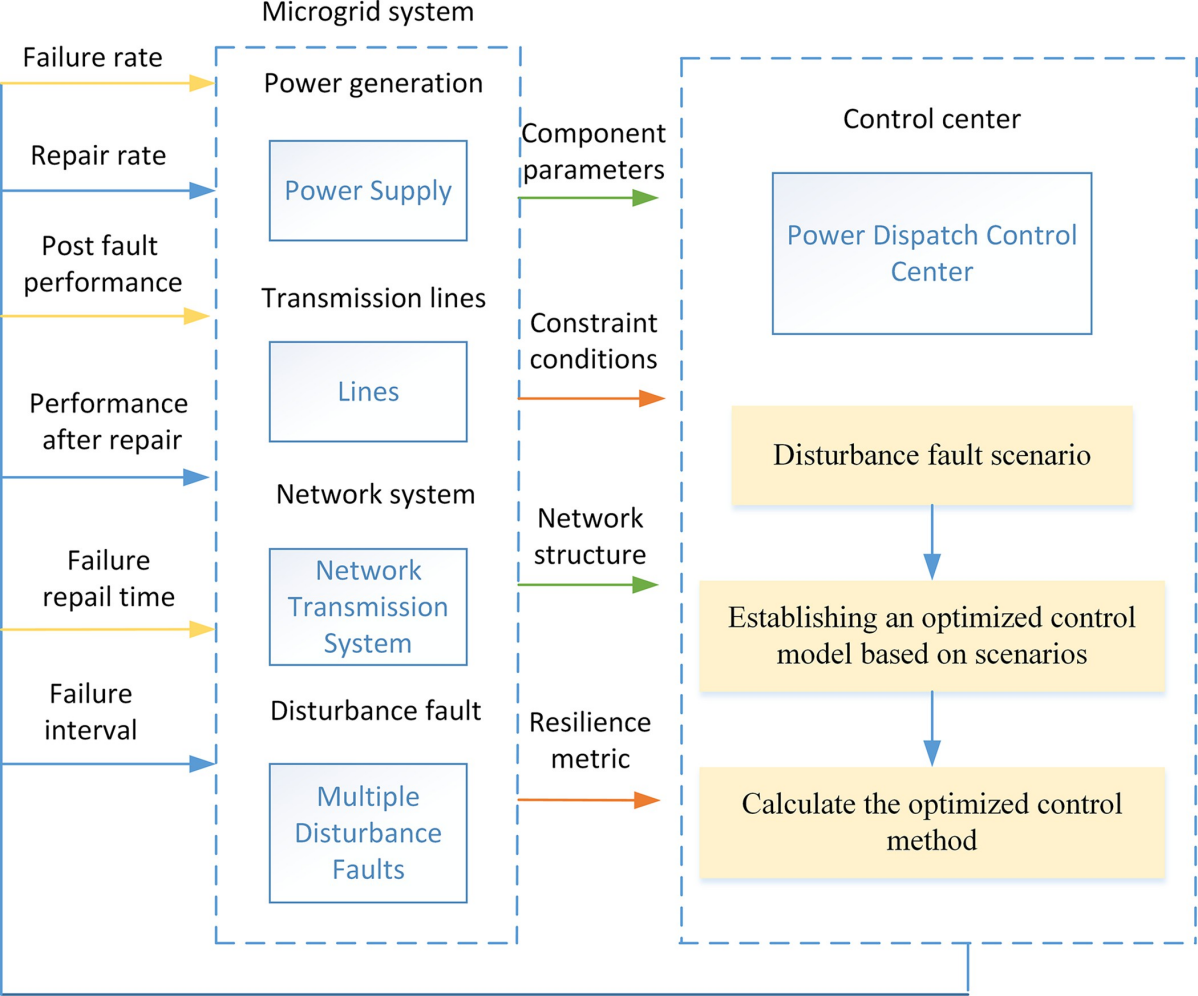
Operation framework of microgrid systems resilience analysis.

Based on the above analysis, the overall analysis process for microgrid system resilience metrics and improvement strategies is as follows. The framework for resilient operation and improvement of microgrid systems is shown in Fig 6.

Step 1: Establish a microgrid system model and collect the required information about the microgrid system components.

Step 2: Establish the parameter information of each component of the microgrid system and send the data information to the control center.

Step 3: Construct a resilience measurement analysis model for the microgrid system, as shown in Eq (4), and calculate the resilience. At the same time, calculate the importance of each component, as shown in Eq (10).

Step 4: Calculate the sensitivity of each parameter based on the information of system components, as shown in Eq (6).

Step 5: Construct an optimization model for the resilience improvement strategy of the microgrid system, as shown in Eq (8), and perform optimization calculations based on the control parameters of each component to obtain an optimization method for resilience improvement. The control center sends control instructions to implement the resilience improvement method.

## 6. Numerical example analysis

### 6.1. Example scenario

**Fig 7:**
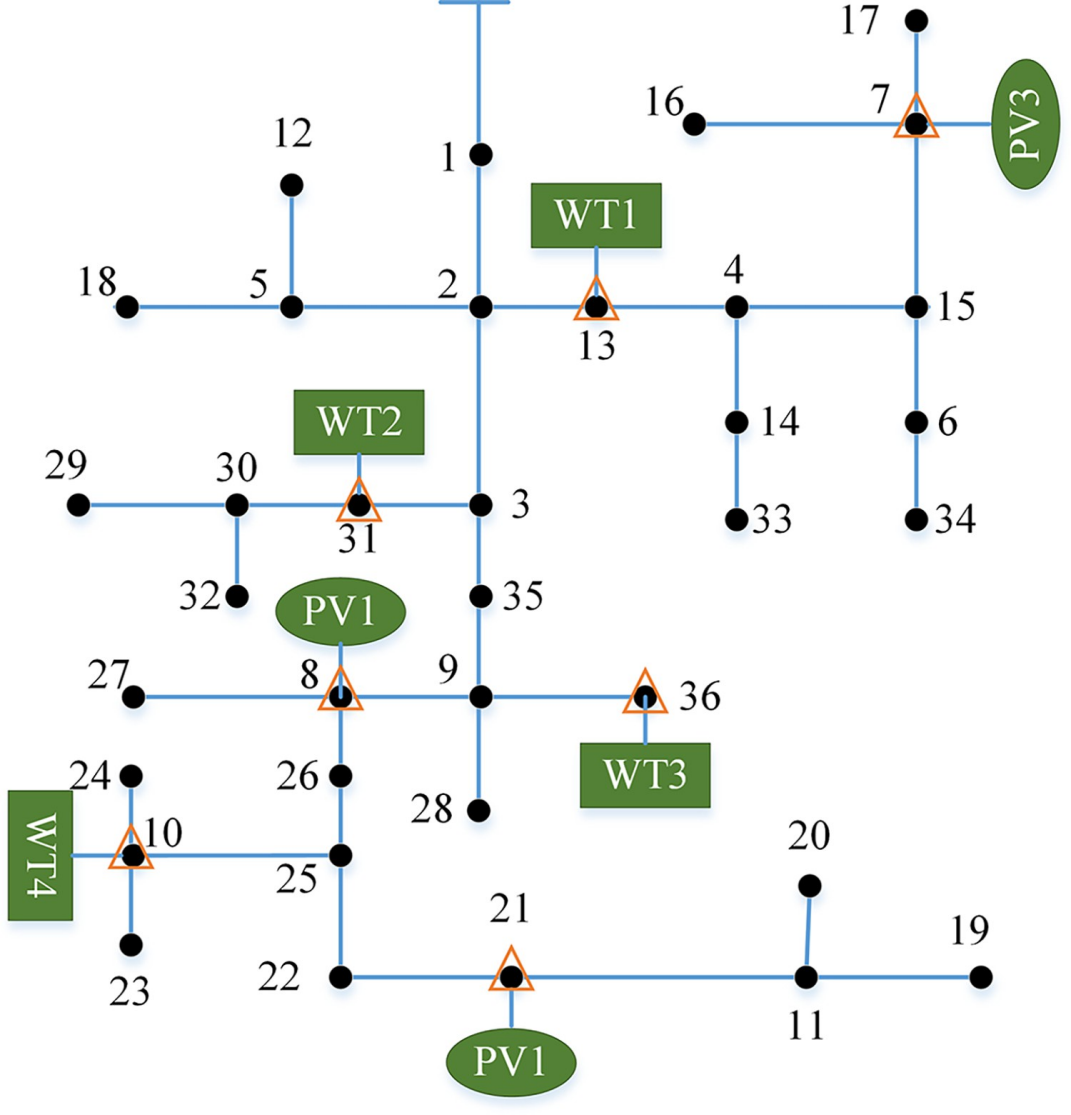
IEEE37-node microgrid system structure diagram.

This article uses the IEEE37-node system as the foundation to construct a microgrid system, and sets up seven new energy injection nodes, namely nodes 7, 8, 10, 13, 21, 31and 36, to verify and analyze the proposed microgrid system resilience metrics and control strategies for enhancing resilience. The framework diagram of the IEEE37-node microgrid system is shown in Fig 7.

In the system shown in Fig 7, there are seven new energy power nodes, including nodes 10, 13, 31 and 36 for wind power generation, and nodes 7, 8 and 21 for photovoltaic power generation. The power generation parameters are shown in Table 1.

**Table 1:** Wind and photovoltaic power generation parameters.

Order number	Network connection point	Maximum active power/kW	Maximum reactive power/kvar
WT1	13	200	120
WT2	31	180	100
WT3	36	160	100
WT4	10	130	110
PV1	8	150	100
PV2	21	80	50
PV3	7	100	80

The parameters of some components of the IEEE37-node microgrid system are shown in Table 2 below.

**Table 2:** Original parameters of some components in the microgrid system.

Components	$P_{f}^{i}$/%	$P_{r}^{i}$/%	$T_{1}^{i}$/h	$T_{2}^{i}$/h
Node 1	2	100	5	240
Node 2	3	100	6	260
Node 5	2	100	4	250
Node 6	1.5	100	6	280
Node 8	2	100	8	260
Node 13	3	100	7	220
Node 21	3	100	8	200
Node 31	2	100	8	280
Node 36	4	100	9	240
Line 1–2	0.2	100	3	300
Line 2–13	0.2	100	5	350
Line 3–31	0.4	100	5	360
Line 8–26	0.4	100	4	360
Line 9–36	0.3	100	3	300
Line 11–21	0.4	100	4	360

To analyze and verify the effectiveness of the proposed method for measuring the resilience of microgrid systems and the resilience improvement strategies, this section sets up two different operating cases for comparative analysis and verification.

Case1. The microgrid system is running normally and has not experienced any malfunction

Case 2. Line fault occurs in the microgrid system. At the same time, based on this, control measures to enhance the resilience of the network system would be taken.

### 6.2. Analysis of calculation results

Case 1. The microgrid system is running normally and has not experienced any malfunction

**Fig 8:**
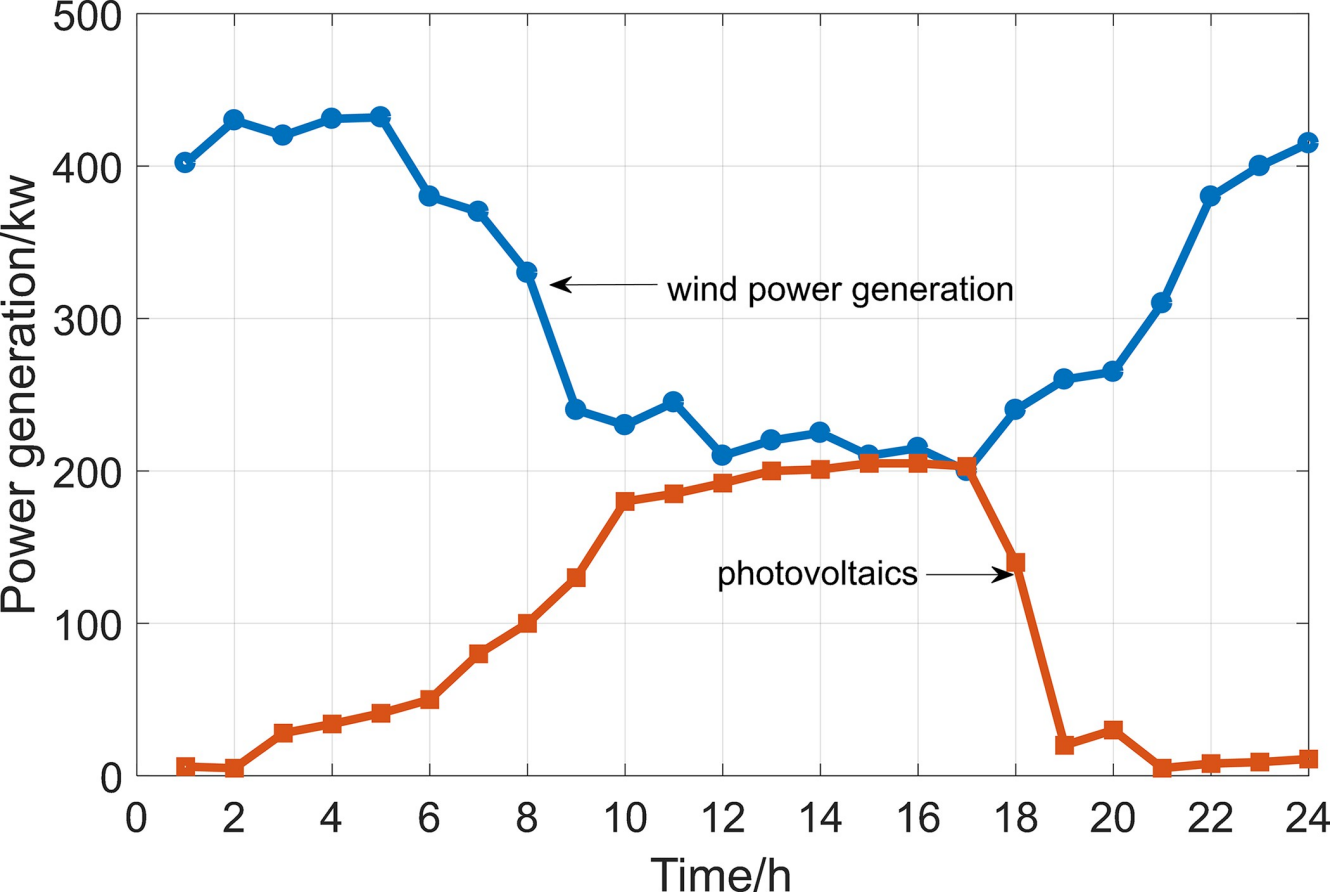
Output of wind power and photovoltaic power generation.

Under case 1, the components of the microgrid system operate normally and there are no faults. The output of wind power and photovoltaic power generation during different periods of time from 0 to 24 hours is shown in Fig 8.

**Fig 9:**
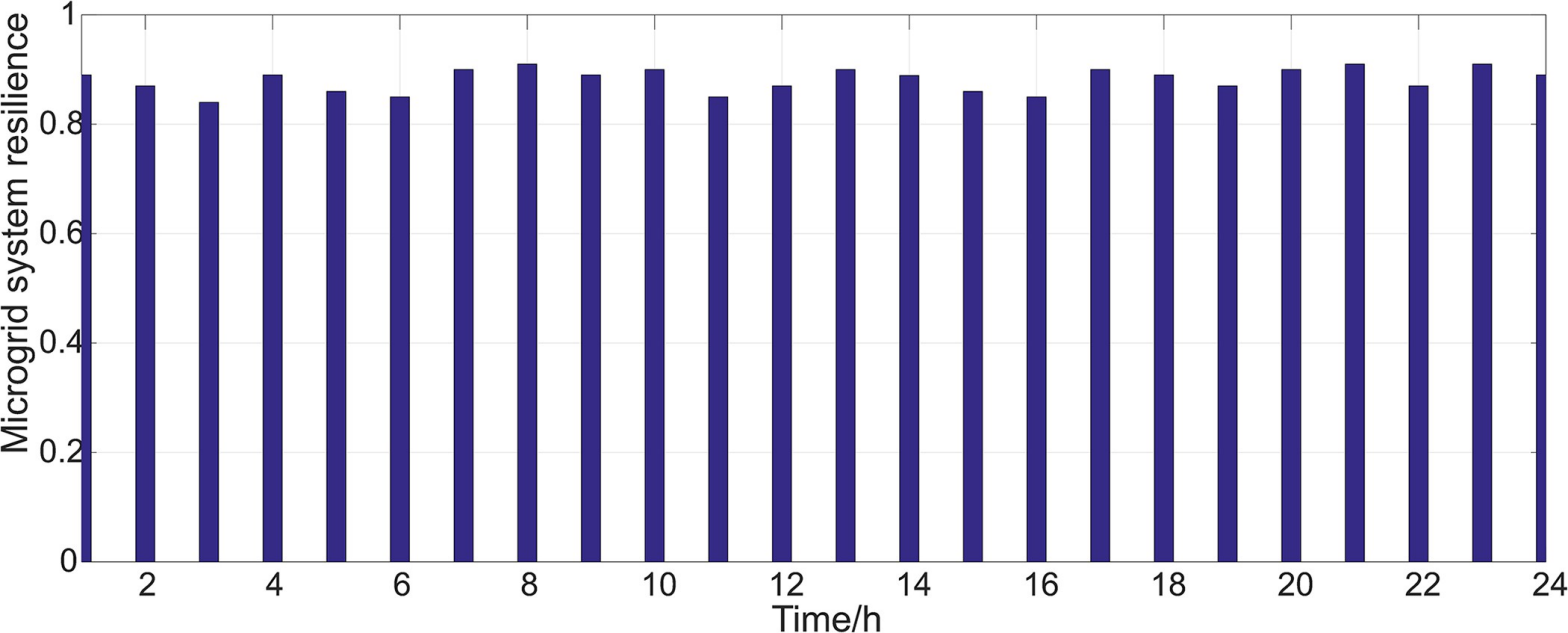
Resilience of microgrid system under case 1.

Using the proposed method for evaluating the resilience of microgrid systems, the resilience of microgrid systems in different time periods can be calculated using Eq (4), as shown in Fig 9.

As shown in Fig 9, when the microgrid is operating normally, the operating status of each component is normal, and the system resilience remains at a roughly stable level.

Case 2. Line fault occurs in the microgrid system

**Fig 10:**
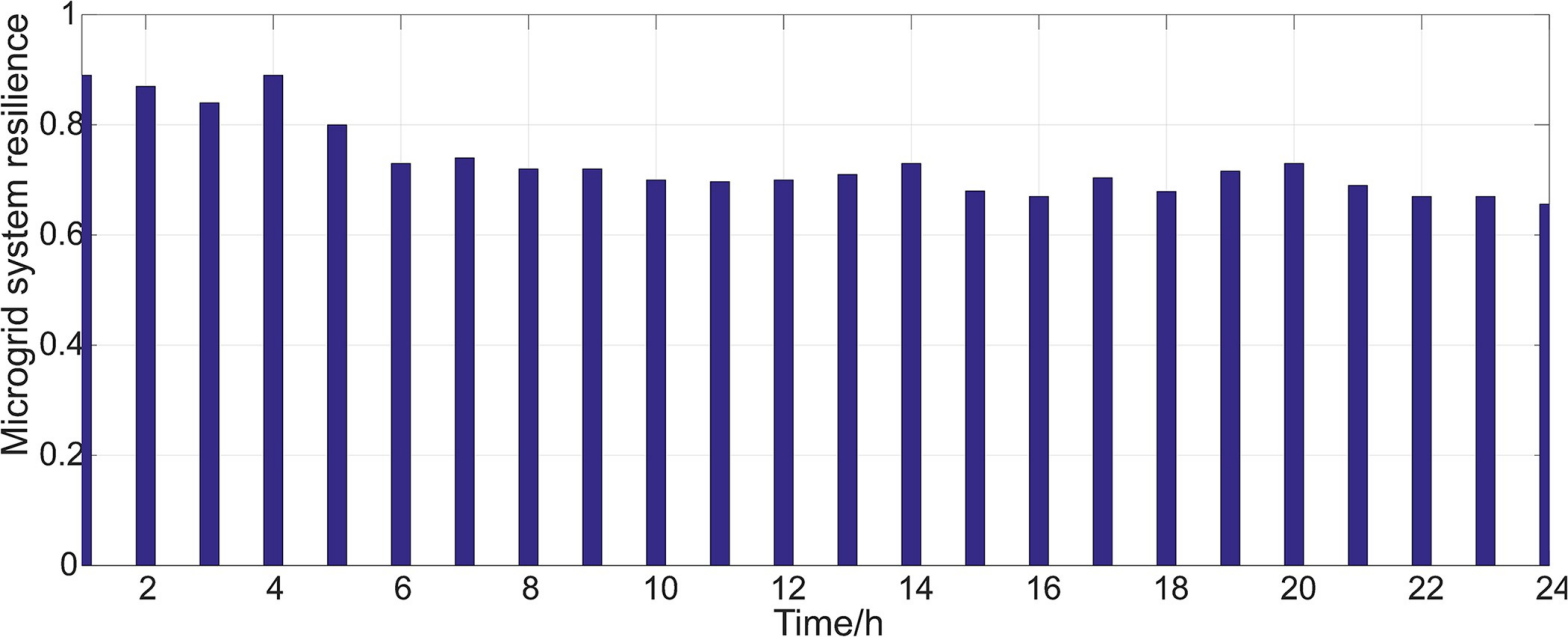
Microgrid system resilience under case 2.

At 4 o’clock, the microgrid system suffered from rainstorm, which led to line 9–36 failure. The parameters of line elements in this section are shown in Table 2. According to formula (4), the resilience in different time periods is shown in Fig 10.

In Fig 10, the resilience of the microgrid system has decreased to some extent. Compared to the normal operation in Fig 9, the microgrid has been affected by natural disasters and weather, resulting in a certain degree of decrease in system resilience.

Therefore, on the basis of Case 2, control measures to enhance the resilience of the network system are taken. The parameters of some line components are adjusted to achieve the goal of enhancing resilience. In the calculation, the range of variation of some control parameters is shown in Table 3.

**Table 3:** The range of some control parameters.

Components	$P_{f}^{i}$/%	$P_{r}^{i}$/%	$T_{1}^{i}$/h	$T_{2}^{i}$/h
Line 8–26	0~0.25	100%	0~2	0~500
Line 9–36	0~0.15	100%	0~2	0~520
Line 11–21	0~0.15	100%	0~2	0~450

Through the calculation of the established optimization control model (8), the adjusted parameters of some components are shown in Table 4.

**Table 4:** Parameters of some components of the microgrid system after regulation.

Components	$P_{f}^{i}$/%	$P_{r}^{i}$/%	$T_{1}^{i}$/h	$T_{2}^{i}$/h
Line 8–26	0.21	100	1.8	450
Line 9–36	0.13	100	1.2	522
Line 11–21	0.16	100	1.78	421

**Fig 11:**
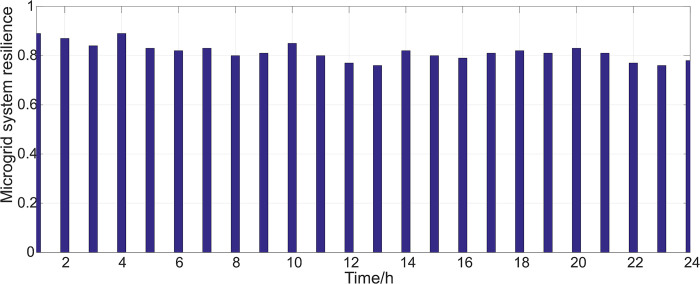
Resilience of microgrid system after optimization and regulation.

By comparing the parameters in Tables 2 and 3, after optimization and adjustment, the component parameters have undergone significant changes, with both the average repair time and the fault interval time being reduced, consistent with actual operating conditions. The resilience calculation results of the microgrid system after optimization and control are shown in Fig 11.

By comparing the calculation results of Figs 10 and 11, it can be seen that after optimization control, the resilience of the microgrid system has been significantly improved, with an increase of more than 10%, which has improved the operation status of the microgrid system to a certain extent and greatly enhanced the system’s operational safety and power supply reliability. This also shows the effectiveness of the resilience improvement strategy proposed in this paper.

In addition, to further verify and analyze the effectiveness of the resilience improvement strategy proposed in this article, a comparative analysis was conducted with existing methods [31], and the operating conditions were set as: a fault occurred on lines 9–36. According to the given resilience recovery strategy method [31], the optimization objective for strategy is to minimize the duration time in emergency recovery phase. If a line fails, the transmission power is zero; If there is no fault in a certain line, the transmission power operates within the limit of rated power. After implementing the power transmission optimization scheme, perform resilience evaluation and compare it with the method proposed in this article. This section compares the differences between different methods from three dimensions: measuring the resilience of microgrid systems, computing time of algorithms, and overall performance of microgrid systems.

**Fig 12:**
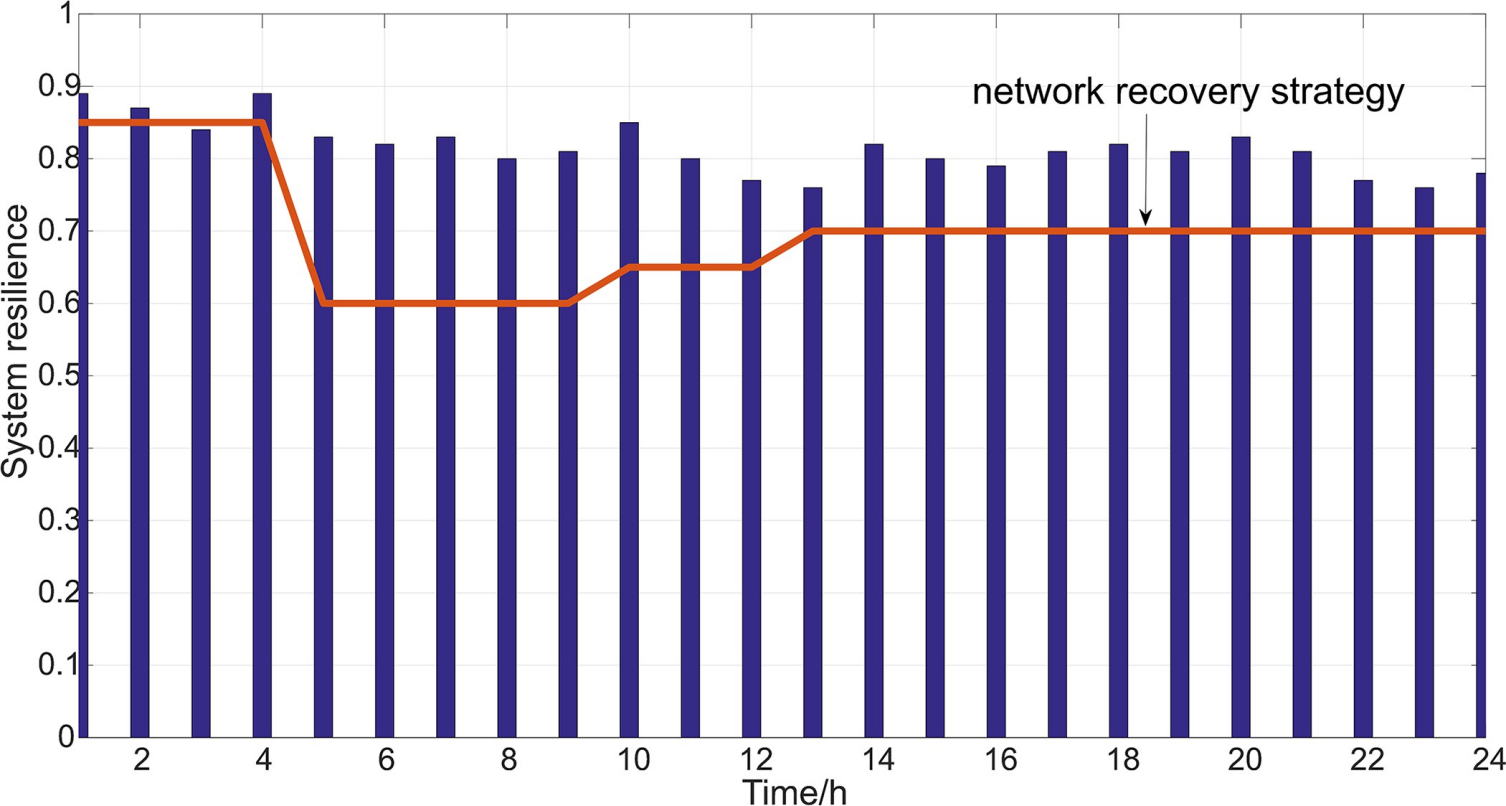
Resilience of microgrid system after optimization and regulation.

As shown in Fig 12 and Table 5, respectively. Under the same conditions as operating case 2, the effect of resilience enhancement was analyzed. According to the method provided in reference [31], the system’s resilience recovery performance was shown in Fig 12.

**Table 5:** Calculation time for different methods.

Methods	Time/s
Network recovery strategy	145
Proposed method in this article	108

The red line in Fig 12 represents the application of network recovery strategy method [31]. After the system is disturbed, the resilience is restored to a certain extent, and the resilience is ultimately improved to 0.7. By applying the method proposed in this article, the system resilience has been restored to around 0.78. The calculation time for two different methods is shown in Table 5 below.

As shown in Table 5, the resilience optimization control strategy proposed in this article is more concise and convenient to implement, with less computation time. Therefore, from the above comparative calculations, it can be seen that the network system resilience optimization and control strategy proposed in this article has shown certain advantages.

**Fig 13:**
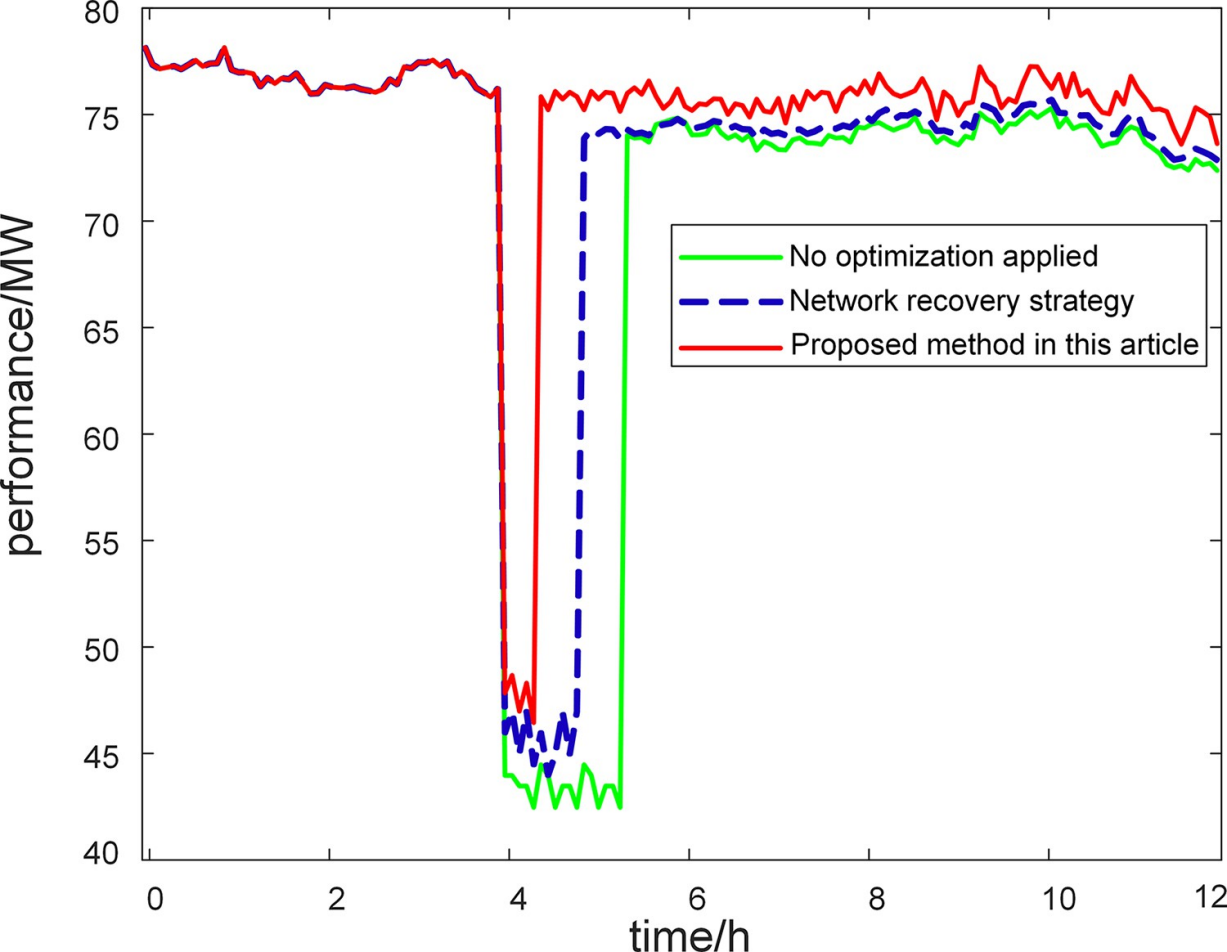
Simulation results of microgrid system performance.

After being disturbed, the overall transmission power performance of the system is shown in Fig 13. Fig 13 shows that the recovery speed of the system varies under different conditions. When a fault disturbance occurs, the transmission power drops to a low value and gradually recovers after the disturbance ends. The calculation results from the graph indicate that if no control and optimization measures are applied to the microgrid system, the system performance recovery will be slower, and it will take approximately 1.3 hours to recover the system performance; The network recovery strategy control method allows for faster system performance recovery, which takes 0.8 hours. Applying the optimization strategy method proposed in this article to regulate the parameters of microgrid system components, the system performance recovers the fastest, only takes 0.4 hours to recover to normal operating state, and achieves the best performance. The comparison of different methods also reveals that the proposed microgrid system resilience improvement strategy has a better effect. Overall, the recovery ability of microgrid system performance is related to the internal component parameters of the system, that is, adopting reasonable parameter optimization and control measures can to some extent restore the system to normal operating state.

### 6.3. Identification of component importance in microgrid system

**Fig 14:**
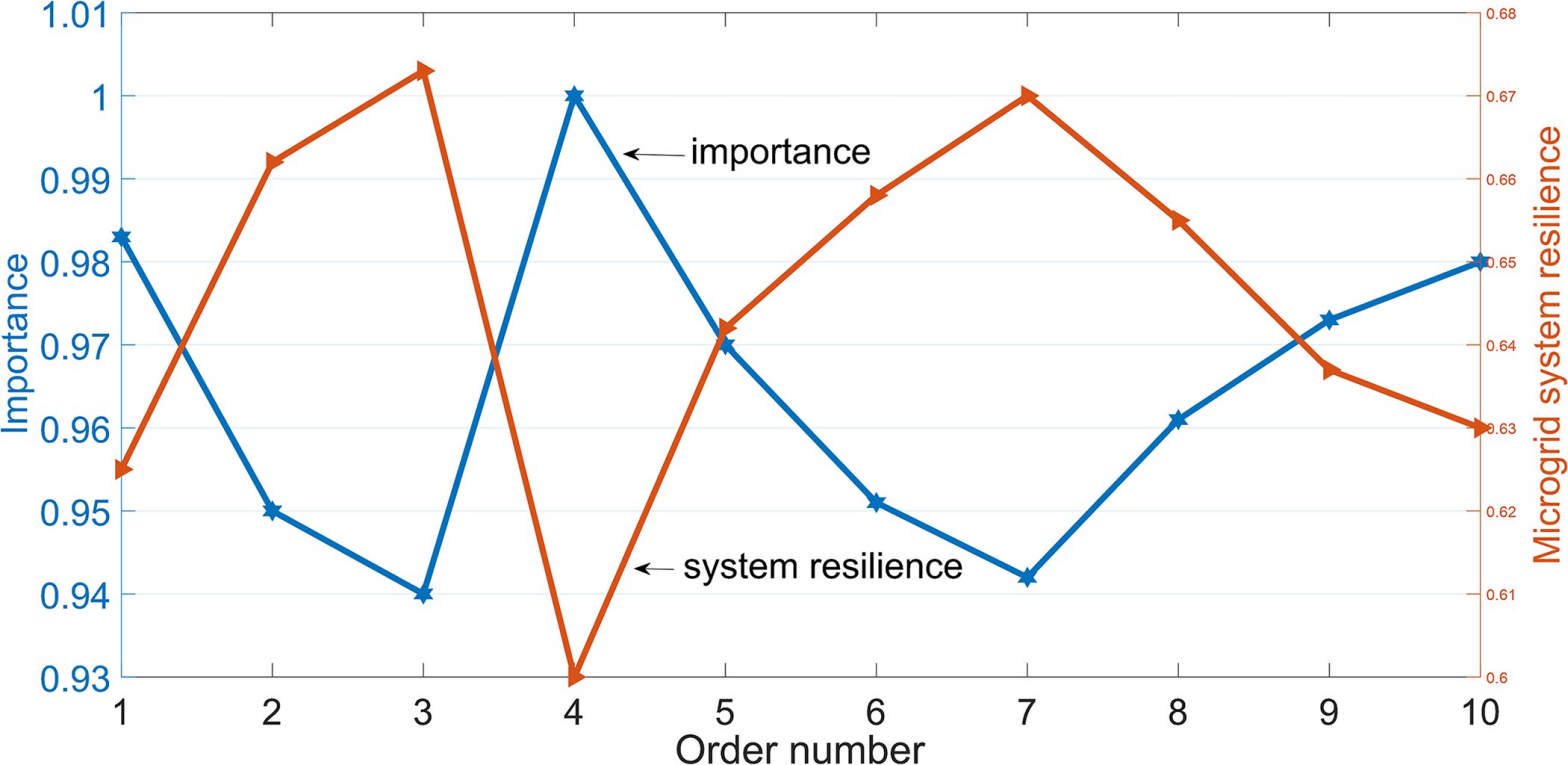
Microgrid system resilience.

The component importance evaluation method given by Eq (10) calculates the importance of components that have failed and cannot be repaired. Some of the calculation results are shown in Table 6. The component importance and corresponding resilience calculation results for the microgrid system in Table 6 are shown in Fig 14.

**Table 6:** Normalized partial component importance.

Order number	Component	Component importance
1	Node 2	0.983
2	Line 3–35	0.95
3	Line 8–26	0.94
4	Node 1	1
5	Node 31	0.97
6	Line 4–15	0.951
7	Line 1–2	0.942
8	Node 8	0.961
9	Node 4	0.973
10	Line 2–3	0.98

Table 6 shows the calculation results of the importance of some components in the microgrid system, and Fig 14 shows the calculation results of the resilience of the microgrid system under the condition of corresponding component failure. For this complex microgrid, nodes 1 and lines 1–2 have high importance. From the network topology structure, it can be seen that the large power grid is connected to the microgrid system through node 1, and line 1–2 is the only branch connecting the large power grid. If node 1 or line 1–2 fails, it will seriously affect the entire microgrid system. In addition, node 4 and node 31 are important nodes in the microgrid system, providing power to adjacent nodes. If node 4 or node 31 fails, it will seriously affect the load power supply of adjacent nodes. The calculation results in Fig 14 show that if the component importance value is larger, the failure will lead to lower resilience of the microgrid system, which is consistent with the actual situation.

It can also be seen that using the resilient calculation method and component importance analysis method proposed in this article can reasonably reflect the ability of the system to respond to multiple disturbances and the importance of components.

## 7. Conclusions

Based on the operating characteristics of microgrid system components, using parameters such as failure rate and failure repair time, considering wind power and photovoltaic grid connection, this paper proposes a resilience analysis and improvement strategy for microgrid systems. The main conclusions are as follows.

Based on the analysis of the resilience of simple microgrid systems, a method for analyzing the resilience of complex microgrid systems considering parameters such as failure rate, repair rate, post-failure performance, post-repair performance, repair time, and average time between failures was proposed. This metric method can better describe the performance maintenance and repair capabilities of microgrid systems under disturbances. Based on the parameters involved in the analysis, the influence of different parameters on system resilience was studied through first-order sensitivity analysis method. Some parameter changes had a positive effect on system resilience, such as repair rate. Some parameter changes had a negative impact on system resilience, such as fault repair time. On this basis, the article proposed an optimization strategy to enhance system resilience, while also considering the economic benefits of adjusting parameters. The results of the example shown that the optimization strategy can effectively improve the level of system resilience.

The article analyzed the representation method of the importance of microgrid system components, which represented the importance of different components in the system on the system’s resilience. The calculation results of numerical examples also demonstrated the rationality of the proposed method.

In this study, it is assumed that the repair rate of the microgrid system is 100% after a failure occurs. If the repair rate is less than 100% or there are other reasons that the failure cannot be fully repaired, the analysis method and improvement strategy for the resilience of the microgrid system will change. Next, we will conduct more in-depth research on the theoretical and technical methods for this situation.

## Supporting information

S1 Data (XLSX)
